# Development of Software Sensors for Determining Total Phosphorus and Total Nitrogen in Waters

**DOI:** 10.3390/ijerph10010219

**Published:** 2013-01-10

**Authors:** Eunhyoung Lee, Sanghoon Han, Hyunook Kim

**Affiliations:** 1 M-Cubic Co., Ltd. Migun Technoworld, 533 Yongsan-dong, Yuseong-gu, Daejeon, 305-500, Korea; E-Mails: lehmmm@empal.com (E.L.); sanghoonhan9@gmail.com (S.H.); 2 Department of Environmental Engineering, University of Seoul, 90 Jeonnong-dong, Dongdaemun-gu, Seoul 130-743, Korea

**Keywords:** software sensor, total nitrogen, total phosphorus, multiple linear regression

## Abstract

Total nitrogen (TN) and total phosphorus (TP) concentrations are important parameters to assess the quality of water bodies and are used as criteria to regulate the water quality of the effluent from a wastewater treatment plant (WWTP) in Korea. Therefore, continuous monitoring of TN and TP using *in situ* instruments is conducted nationwide in Korea. However, most *in situ* instruments in the market are expensive and require a time-consuming sample pretreatment step, which hinders the widespread use of *in situ* TN and TP monitoring. In this study, therefore, software sensors based on multiple-regression with a few easily *in situ* measurable water quality parameters were applied to estimate the TN and TP concentrations in a stream, a lake, combined sewer overflows (CSOs), and WWTP effluent. In general, the developed software sensors predicted TN and TP concentrations of the WWTP effluent and CSOs reasonably well. However, they showed relatively lower predictability for TN and TP concentrations of stream and lake waters, possibly because the water quality of stream and lake waters is more variable than that of WWTP effluent or CSOs.

## 1. Introduction

The Korean Ministry of Environment has recently imposed stricter permit requirement on the outflow of domestic wastewater treatment plants (WWTPs) to improve the water quality of receiving water bodies such as rivers and lakes. Therefore, the water quality monitoring program has become an important social issue. 

At present, there are a total of 61 *in situ* monitoring stations along the banks of major streams and lakes to measure the status of the water quality on-site. In addition, since 2008, a total of 653 tele-metering systems have been installed at the discharge point of each of medium to large size WWTP for monitoring effluent water quality continuously. The water quality parameters monitored by the systems include pH, dissolved oxygen (DO), electrical conductivity (EC), turbidity (Turb), chemical oxygen demand (COD), total nitrogen (TN), and total phosphorus (TP). Among these parameters, TN and TP are the most important ones and obligatory parameters, and are monitored using automated laboratory instruments, which are as expensive as 100,000 USD each. Moreover, these instruments require time-consuming sample pretreatment before water TN and TP are determined (usually more than 1 h), which hinders the widespread use of *in situ* monitoring of TN and TP. 

A software sensor is a common name for the software in which a given set of water quality data obtainable by easy and reliable methods are processed to estimate the quantities of other water quality variables using a model [1,2]. In general, a variable that cannot be easily measurable is selected as the one estimated by the software sensor. It is normally developed in a form of statistical models such as a multiple linear regression (MLR) model.

**Figure 1:**
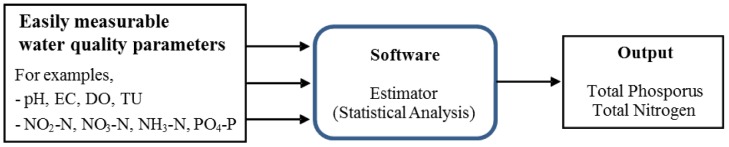
Concept of software sensor.

The basic concept of the software sensor is illustrated in Figure 1. Measurement values for water quality parameters that can be relatively easily measurable are fed into a software sensor (called an estimator) and are processed to provide other water quality parameters, for examples, TN or TP [3,4]. Using software sensors, it is possible to create continuous time series of TP and TN data that can be utilized for better understanding the timing and magnitude of TP and TN fluxes to streams or lakes.

In fact, the software sensor concept has been applied in a few studies. Christensen *et al.* [5,6] developed MLR based software sensors to predict total suspended solids (TSS), fecal coliforms, and nutrients for several streams in Kansas, USA, using real-time measured Turb, specific conductance, water temperature, and discharge. Data from the software sensor was applied to calculate total maximum loads of the TSS on the streams. Uhrich *et al.* [7] derived power regression equations for estimating suspended-sediment concentrations from instream real-time Turb-monitor data in the upper North Santian river basin, Oregon, USA. Zhu *et al.* [8] also applied an MLR-based software sensor for the prediction of stream flow and runoff in Pennsylvania, USA, using geographic information system. 

The software sensor concept also has been applied in WWTPs. Alastair *et al.* [9] estimated bicarbonate alkalinity using a MLR model based on pH, redox and conductivity data to control actuators in the anaerobic digestion process. In a study carried out by Alcaraz-González *et al.* [10], flow rate, CO_2_ exhaust flow rate, fatty acid concentration and total inorganic carbon were utilized to estimate microbial concentrations, alkalinity and COD in each unit processes of a WWTP. Lastly, Feitkenhauer and Meyer [11] estimated substrate and biomass concentrations and controlled aerobic cycle of aerobic and anoxic activated sludge process using a titrimetric technique based software sensor.

Total nitrogen and TP in streams or wastewater have been measured using software sensors by a few researchers. Jeong *et al.* [12] tried to measure TN and TP in wastewater *in situ* using UV absorbance and an artificial neural network (ANN)-based model. da Costa *et al.* [13] used an ANN model to predict TN and PO_4_^3−^ concentrations of streams. In their study, however, the ANN model was fed with data from *in situ *surrogate sensors, *i.e.*, temperature, pH, DO, and EC sensors. Ryberg [14] and Christensen *et al.* [15] applied MLR models fed with data from *in situ* stream flow, EC, pH, temperature, Turb, and DO sensors for predicting TN and TP of streams. Even with the data from surrogate sensors, their models could reasonably predict the TN and TP of their streams; R^2^ s of the MLR models for TN and TP were 0.70, and 0.77, respectively. 

In this study, software sensors (or regression models) were developed to estimate TN and TP of different waters (*i.e.*, streams, lakes, WWTP effluents, and CSOs) by performing MLR with water quality parameters including pH, EC, DO, Turb, NO_2_–N, NO_3_–N, NH_4_–N, and PO_4_–P. This study was intended to evaluate the feasibility of the software sensor concept in indirect measurement of TN and TP in waters. Moreover, in this study, ionic nutrient species data were also included in the MLR models, so a better model performance was expected.

## 2. Materials and Methods

### 2.1. Study Area and Data Acquisition

**Figure 2:**
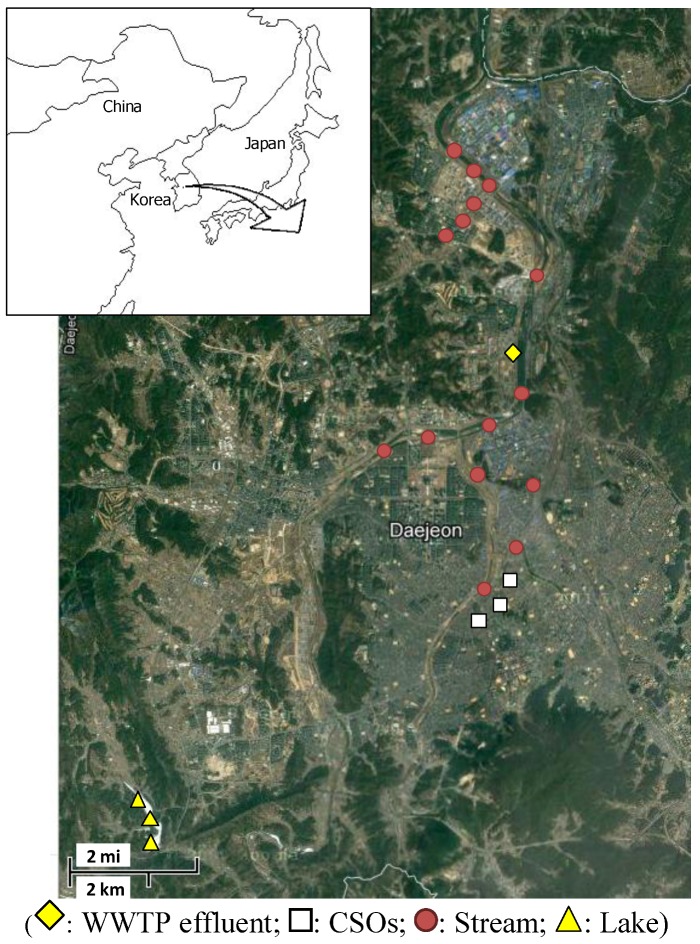
Water sampling locations.

Water samples for the current study were collected from the Daejeon area in the middle of South Korea (Figure 2). Water samples were collected from a total of 22 points; 15 points for stream water samples, three for lake water, three for CSOs, and one for WWTP effluent. The predictability of a software sensor may be improved if water qualities are measured at other points in a WWTP. However, the water quality of only the outflow from a WWTP is under surveillance in Korea. Therefore, in this study, we just focused on the outflow site only. The WWTP is treating domestic wastewater and is consisted of a conventional activated sludge process and a subsequent coagulation process for phosphorus removal. The stream under study is flowing along the urban area and receiving treated wastewater from the WWTP. Finally, the lake is located in the upstream of the agricultural and forestry area. The lake water samples were collected from about 0.5 m depth from the surface.

Water samples were collected weekly from March, 2011 to June, 2012. In Table 1 and Table 2, the number of water samples collected for each water type and the water quality parameters analyzed in the laboratory are summarized, respectively. For the study, the whole observation data were divided into two sets; one for calibration (or training) and the other for validation.

Namely, water quality data collected from March 2011 to August 2011 were used for model development, and the data from September 2011 to June 2012 were used for model validation.

**Table 1:** Conditions of water quality analysis.

Water Type	Sampling points	Number of samples
WWTP effluent	1	77
CSOs	3	239
Streams	15	228
Lakes	3	1,183

All the water quality parameters except TP and TN in Table 2 were used as independent variables in the MLR analysis: input data for a software sensor (or a regression model). The manually measured TN and TP concentrations were compared with the ones predicted by the developed software sensors. DO, pH, EC, and Turb were measured using a sensor (YSI6600EDS SONDE, YSI Inc., Yellow Springs, OH, USA), while NO_2_–N, NO_3_–N, NH_4_–N, and PO_4_–P were done with ion chromatography (IC; DIONEX-ICS-1100, Thermo-Fisher Inc., Seoul, Korea).

**Table 2:** Water quality parameters monitored in this study.

	Water quality Parameters	Unit	Measurement Method
Variables measured by sensors	DO	mg·L^−1^	Electrode Method (YSI6600EDS SONDE)
	pH	-	
	EC	μS·cm^−1^	
	Turb	NTU	
Variables measured by chemical analysis	PO_4_–P	mg·L^−1^	IC (DIONEX-ICS-1100)
	NO_2_–N	mg·L^−1^	
	NO_3_–N	mg·L^−1^	
	NH_4_–N	mg·L^−1^	
	TP	mg·L^−1^	Ascorbic Acid Method
	TN	mg·L^−1^	Persulfate Method

### 2.2. Data Processing

#### 2.2.1. Scatter Diagram Analysis

Initially, the correlation between different water quality parameters was analyzed. For better understanding the relationship, a scatter diagram was first drawn for pairs between TN or TP and each of the other water quality parameters. A scatter diagram can visually show the relative strength of the relationship between each pair of variables; the direction (*i.e.*, positively or negatively correlated) and shape (*i.e.*, linear or non-linear) of the correlation can be shown. The scatter diagram shows to what extent each water quality parameter correlates with TN and TP. The correlation coefficient between two variables is defined as the covariance of the two variables divided by the product of their standard deviations. Out of the scatter diagram analysis, dominant or important parameters can be derived from all the variables; if any parameter is highly correlated with TN or TP, it can be regarded as an important parameter. 

#### 2.2.2. Multiple Linear Regression Analysis

Dominant variables, which were derived as the result of a scatter diagram analysis, were utilized to develop a software sensor to predict TN and TP through the MLR analysis as a next step. An MLR is an analytical method used to develop an equation to relate a dependent variable *y* and one or more independent variables. In fact, an MLR is still used extensively in practical applications. A linear regression model or equation depends on the linear relation between its known and unknown variables, and it is easier to fit than a non-linear model. It is also easier to determine the statistical properties of the resulting estimators (*i.e.*, software sensors or linear models). 

A general MLR equation (or the software sensor in this study) is provided below (Equation (1)):

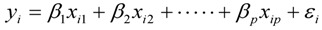
(1)
where *y_i_* is a dependent variable (TN or TP concentration in this study), *x_i_* represents independent variables (water quality parameters other than TN and TP in this study), *β* is a regression coefficient, *p* is the number of independent variables, *n* is number of datasets, and ε is an error term [16]. 

In this study, we applied the stepwise regression based on forward selection. Namely, we started with a model with one explanatory variable that had been identified as the most significant, and added variables one by one until we could not improve the model significantly by adding another variable [17]. However, each time a new variable was added, the significance of each variable in the model was tested. The *p*-value for inclusion of a new variable was set at 0.05 in this study. In addition, if the *p*-value of a variable in the model was higher than a preset threshold (in this study, p < 0.1), it was eliminated. The model was then refitted to the data set, before the next forward selection procedure was performed. This procedure was repeated until the model was not further improved by the addition of any variable. We used the Statistical Package for the Social Sciences (SPSS; IBM, Armonk, NY, USA) for a stepwise MLR analysis to derive significant independent variables among all water quality parameters listed in Table 2 [18]. The predictability of the developed models or software sensors was evaluated using the mean square error (MSE), and the adjusted coefficient of determination (*R_a_*^2^). The MSE is used to assess the variance between measured and estimated values, and the *R_a_*^2^ is the variance fraction of measured values explained by a regression model. 

## 3. Results and Discussion

### 3.1. Water Quality Measurement Data

**Figure 3:**
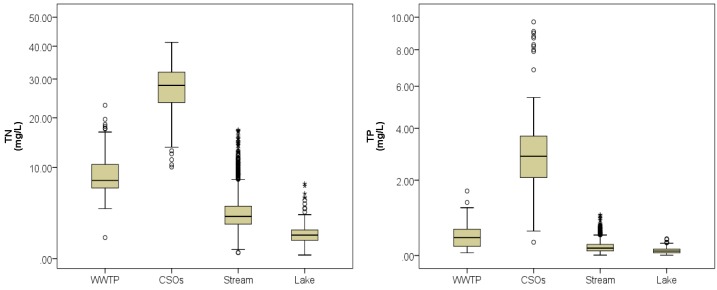
Comparison of water TN and TP concentrations for different water types (circles and stars indicate outliers).

Figure 3 compares TN and TP levels of WWTP effluents, CSOs, stream waters, and lake waters; the statistics of the measurements are summarized in Table 3. 

**Table 3:** TN and TP of water samples from different locations.

Parameters	Type	Min	Max	Mean	Median	Standard deviation
TN	WWTP	1.36	23.01	9.179	7.897	4.188
	CSOs	10.08	41.31	27.415	28.250	6.450
	Stream	0.32	17.30	4.112	3.297	2.747
	Lake	0.19	7.44	1.739	1.549	1.021
TP	WWTP	0.052	1.646	0.445	0.374	0.334
	CSOs	0.274	9.700	3.051	2.855	1.495
	Stream	0.007	0.950	0.176	0.145	0.132
	Lake	0.005	0.350	0.097	0.088	0.062

Both box plots for the TN and TP concentrations of the CSOs have long whiskers indicating the widespread data. Another notable feature is that water quality data for the streams and lakes have a few outliers exceeding 1.5× inter-quartile range, compared with those for other water types [19]. This indicates that natural water (*i.e.*, stream or lake water) is quite variable and vulnerable to weather conditions or other external nutrient sources. These water quality changes of a river and a lake were expected to affect the model performance.

### 3.2. Result of Scatter Diagram Analysis

**Figure 4:**
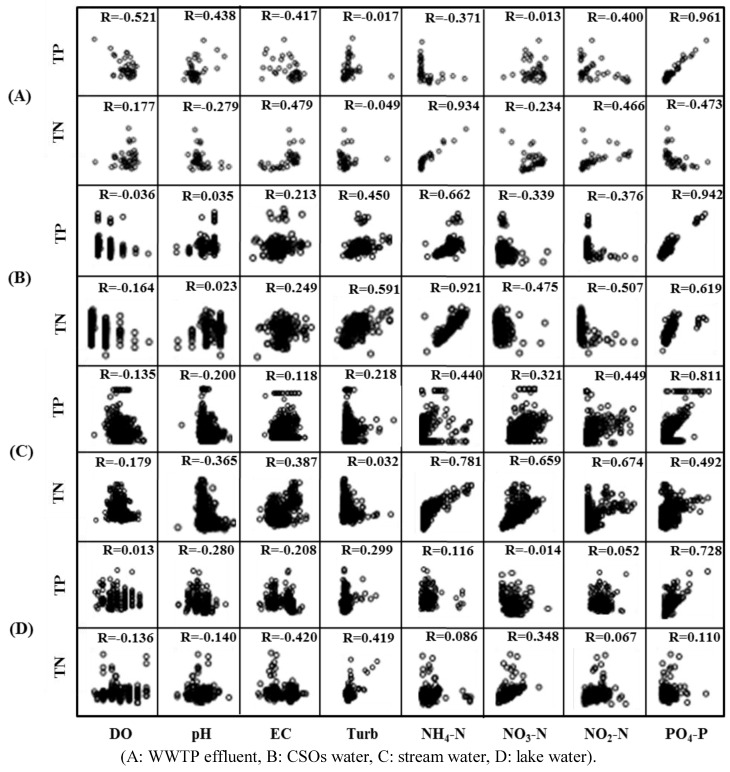
Scatter plots of water quality parameters for four water types.

The scatter plots constructed for all data measured from March, 2011 to August, 2011 in this study are shown in Figure 4. The scatter plots visualize the correlation of each pair between TN or TP and one of other water quality parameters. The data of the WWTP effluent show that the TN concentration had a positive correlation with NH_4_–N (r = 0.94) and that the TP concentration also had a good positive correlation with PO_4_–P (r = 0.96). The water quality data for CSOs also show that the TN concentration had a positive correlation with NH_4_–N (r = 0.92) and the TP concentration with PO_4_–P (r = 0.94). In the case of the stream, the TN concentration was positively correlated with NH_4_–N (r = 0.80) and the TP concentration was with PO_4_–P (r = 0.82). However, the data obtained by analyzing lake waters did not show a good correlation between TN or TP and other water quality parameters. Nonetheless, Turb (r = 0.42) and NO_3_–N (r = 0.35) concentrations had a slightly better correlation with the TN of the lake waters. Only PO_4_–P had a good correlation with TP (r = 0.73).

In fact, the relatively lower correlation between TN or TP and other water quality parameters for stream and lake waters was expected. The water qualities of the lake and the stream are often affected by the external pollutant sources, internal reactions, or weather conditions.

Typically, DO, pH and EC data did not show significant correlation with the TN (r = −0.18 − 0.18 for DO, r = −0.37 − 0.02 for pH and r = −0.42 − 0.48 for EC) or the TP concentrations (r = −0.52 − 0.01 for DO, r = −0.28 − 0.44 for pH and r = −0.42 − 0.21 for EC) for all water types. 

### 3.3. Multiple Linear Regression Analysis for Each Water Types

#### 3.3.1. MLR Analysis for WWTP Effluent

With the datasets for the WWTP effluent, the stepwise MLR analysis was conducted. The result of the regression analysis is summarized in Table 4. For the MRL analysis, the TN and TP concentrations were set as dependent variables, and the most dominant parameters were initially considered as the only independent variable for each regression model, with other significant independent variables added one by one. As the number of independent variables increased from 1 to 3 in the model for the TN estimation, the *R_a_*^2^ value also increased gradually. However, if one of the other variables which did not have a good correlation with the TN was added, the *R_a_*^2^ value of the regression was deteriorated.

Model_N_-3 for estimating TN in Table 4 showed the best fit to the measured TN data (*R_a_*^2^ = 0.978), while Model_P_-1 for estimating TP, which included only PO_4_^3−^–P data as independent variable showed the best fit to the measured TP data (*R_a_*^2^ = 0.936). In short, as a result of these analyses, it was concluded that the TN and TP concentrations of the WWTP effluent are feasible parameters that can be estimated using a software sensor. This is mainly due to the fact that the water quality of the WWTP discharge is relatively stable, compared with natural waters. In fact, the effluent water quality of a WWTP does not change much as long as the WWTP is operated at steady state. In addition, the high degree of correlation between PO_4_–P and TP in the WWTP effluent indicates that most of the phosphorus species in the effluent were in the dissolved form rather than in particulate ones.

**Table 4:** Variance analysis of models predicting TN and TP of WWTP effluent.

TN (Dependent variable)				TP (Dependent variable)			
Model	Mean square	*R_a_*^2^	*p*-value	Model	Mean square	*R_a_*^2^	*p*-value
Model_N_-1 ^a^	552.371	0.882	<0.01	Model_P_-1 ^a^	4.582	0.936	<0.01
Model_N_-2 ^b^	305.321	0.975	<0.01				
Model_N_-3 ^c^	204.081	0.978	<0.01				
Independent variables				Independent variables			
a NH_4_–N				a PO_4_–P			
b NH_4_–N, NO_3_–N							
c NH_4_–N, NO_3_–N, PO_4_–P							

#### 3.3.2. MLR Analysis for CSOs Water

With the water quality parameters measured for CSOs waters, the stepwise MLR analysis was conducted. The result of the analysis is summarized in Table 5. 

**Table 5:** Variance analysis of models predicting TN and TP of CSOs.

TN (Dependent variable)				TP (Dependent variable)			
Model	Mean square	*R_a_* ^2^	p-value	Model	Mean square	*R_a_* ^2^	p-value
Model_N_-1 ^a^	3518.589	0.858	<0.01	Model_P_-1 ^a^	325.279	0.902	<0.01
Model_N_-2 ^b^	1781.741	0.869	<0.01	Model_P_-2 ^b^	165.252	0.917	<0.01
Independent variables				Independent variables			
a NH_4_–N				a PO_4_–P			
b NH_4_–N, PO_4_–P				b PO_4_–P, NH_4_–N			

From the scatter plots for the CSOs water, five variables (*i.e.*, NH_4_–N, PO_4_–P, Turb, NO_3_–N, and DO) were found to have significant correlation with the measured TN concentration, while three variables (*i.e.*, PO_4_–P, DO, NO_3_–N) were significantly correlated with the TP concentration. However, the MLR analysis showed that the models with one independent variable (*i.e.*, NH_4_–N) and two (*i.e.*, NH_4_–N and PO_4_–P) showed the best fit to the measured TN. Model_N_-1 with one dependent variable showed the *R_a_*^2^ of 0.858 while Model_N_-2 did the *R_a_*^2^ of 0.869. In the case of models for the prediction of TP, PO_4_–P was identified as the most important variable. The Model_P_-2 which has two variables (*i.e.*, NH_4_–N and PO_4_–P) showed the highest *R_a_*^2^ value (= 0.917). The result showed that the contribution of other variables to the prediction of the TP of CSOs might not be significant. 

#### 3.3.3. MRL Analysis for Stream Water

Using the water quality data for stream waters, a stepwise MRL analysis was carried out. The summary of the analysis is provided in Table 6. Since NH_4_–N was identified as the dominant variable in the estimation of TN concentration, the regression model was expanded from the one with NH_4_-N as the only independent variable to the ones with NO_3_–N, Turb, PO_4_–P, pH, NO_2_–N, and EC in a stepwise manner. In short, Model_N_-7 with NH_4_–N, NO_3_–N, Turb, PO_4_–P, pH, NO_2_–N, and EC as independent variables showed the best fit to the measured TN concentration. Therefore, the model was chosen as the software sensor to estimate TN in stream waters. For the TP concentration, Model_P_-6 showed the best fit to the measured TP data, although the *R_a_*^2^ value was only 0.746; over 70% of the measured data could be explained by the model. One of the major reasons that low *R_a_*^2^ value was obtained might be the low TP concentration of the stream waters; the TP of all the stream water samples was below 1.0 mg·L^−1^ with the majority below 0.5 mg·L^−1^ (Figure 3). At such a low concentration, errors from manual measurements also may contribute to the error from the model predictions. 

**Table 6:** Variance analysis of models predicting TN and TP of stream water.

TN (Dependent variable)				TP (Dependent variable)			
Model	Mean square	*R_a_* ^2^	*p*-value	Model	Mean square	*R_a_* ^2^	*p*-value
Model_N_-1 ^a^	3135.004	0. 633	<0.01	Model_P_-1 ^a^	8.892	0.675	<0.01
Model_N_-2 ^b^	2001.062	0.808	<0.01	Model_P_-2 ^b^	4.759	0.723	<0.01
Model_N_-3 ^c^	1361.633	0.825	<0.01	Model_P_-3 ^c^	3.244	0.739	<0.01
Model_N_-4 ^d^	1026.397	0.829	<0.01	Model_P_-4 ^d^	2.44	0.741	<0.01
Model_N_-5 ^e^	827.979	0.836	<0.01	Model_P_-5 ^e^	1.957	0.743	<0.01
Model_N_-6 ^f^	693.635	0.84	<0.01	Model_P_-6 ^f^	1.636	0.746	<0.01
Independent variables				Independent variables			
a NH_4_–N				a PO_4_–P			
b NH_4_–N, NO_3_–N				b PO_4_–P, Turb			
c NH_4_–N, NO_3_–N, Turb				c PO_4_–P, Turb, NH_4_–N			
d NH_4_–N, NO_3_–N, Turb ,EC,				d PO_4_–P, Turb, NH_4_–N, NO_2_–N			
e NH_4_–N, NO_3_–N, Turb, EC, NO_2_–N,				e PO_4_–P, Turb, NH_4_–N, NO_2_–N, NO_3_–N			
f NH_4_–N, NO_3_–N, Turb, EC, NO_2_–N, pH,				f PO_4_–P, Turb, NH_4_–N, NO_2_–N, NO_3_–N, pH			

#### 3.3.4. MLR Analysis for Lake Water

Using the water quality data for water samples collected from the lake of interest, the stepwise MLR analysis was conducted. The summary of the results is provided in Table 7. Unlike the other cases, any of the models developed through the MRL analyses did not show a good fit to the measured TN. It is because the TN concentration of the lake water was not well correlated with any other water quality parameters (Figure 4). The best fit model for the TN estimation was identified Model_N_-2 with Turb, and NO_3_-N as independent variables (*R_a_*^2^ = 0.417). 

The case for predicting TP concentration was similar to the one for TN. The model with PO_4_–P, EC, and NO_3_-N as independent variables (*i.e.*, Model-3) showed the best fit to the measured TP with the *R_a_*^2^ of 0.612. One thing of interest is that the model with EC as the only independent variable showed a comparable *R_a_*^2^ value with the Model_P_-3, indicating the EC data correlated with the TP concentration.

Again, as the case with the stream waters, the TP concentrations of lake waters was too low; all the data was below 0.5 mg·L^−1^. Therefore, it was hypothesized that errors from manual measurements might affect the overall predictability of the models. 

**Table 7:** Variance analysis of models predicting TN and TP of lake water.

TN(Dependent Variable)				TP(Dependent Variable)			
Model	Mean square	*R_a_* ^2^	*p*-value	Model	Mean square	*R_a_* ^2^	*p-*value
Model_N_-1 ^a^	64.883	0.348	<0.01	Model_P_-1 ^a^	0.305	0.572	<0.01
Model_N_-2 ^b^	38.921	0.417	<0.01	Model_P_-2 ^b^	0.16	0.599	<0.01
				Model_P_-3 ^c^	0.109	0.612	<0.01
Independent variables				Independent variables			
a Turb				a PO_4_–P			
b Turb, NO_3_–N				b PO_4_–P, EC			
				c PO_4_–P, EC, NO_3_–N			

#### 3.3.5. Summary of MRL Analyses for Different Water Types

The best regression models for TN and TP derived from each MLR analysis for each water type are listed in Table 8. 

**Table 8:** Software sensors obtained from MLR analysis.

Sites	Estimated parameters	Correlation equations	*R_a_* ^2^
WWTP effluent	TN	0.881 + 0.986 × NH_4_–N + 1.092 × NO_3_–N + 0.631 × PO_4_–P	0.978
	TP	0.148 + 0.946 × PO_4_–P	0.936
CSOs	TN	5.918 + 0.857 × NH_4_–N + 0.405 × PO_4_–P	0.869
	TP	0.500 + 0.851 × PO_4_–P + 0.04 × NH_4_–N	0.917
Stream water	TN	4.569 + 1.025 × NH_4_–N + 0.838 × NO_3_–N + 0.018 × Turb − 0.004 × EC + 5.432 × NO_2_–N − 0.336 × pH	0.840
	TP	0.171 + 0.964 × PO_4_–P + 0.002 × Turb + 0.008 × NH_4_–N + 0.190 × NO_2_–N − 0.01 × NO_3_–N − 0.013 × pH	0.746
Lake water	TN	0.361 + 0.158 × Turb + 0.693 × NO_3_–N	0.417
	TP	0.158 + 0.962 × PO_4_–P − 0.001 × EC − 0.017×NO_3_–N	0.612

**Figure 5:**
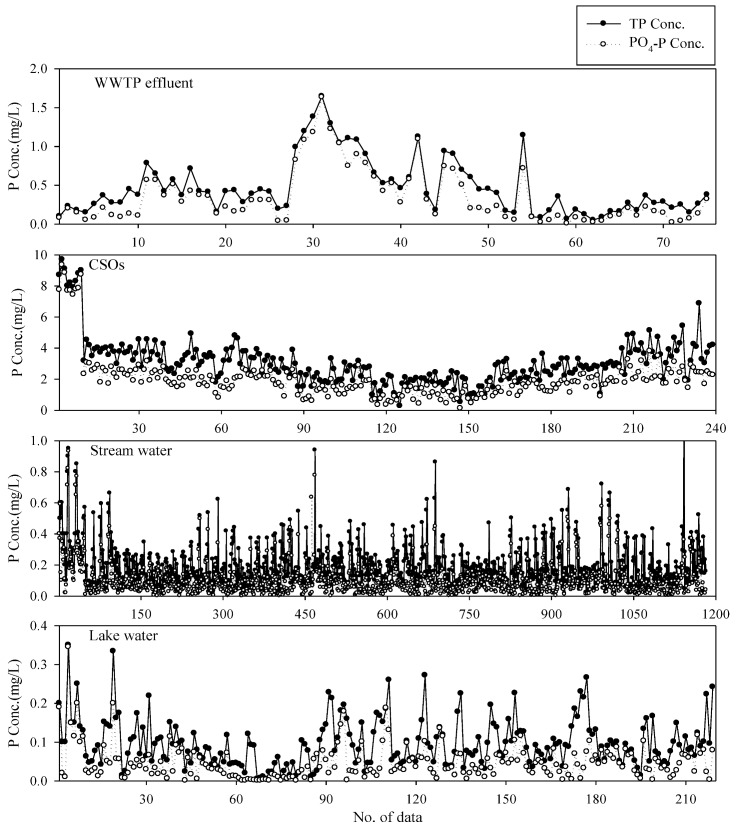
Comparison of PO_4_–P and TP concentrations for each water type.

These regression equations can be used as a software sensor. As stated above, the equations for the WWTP effluent and CSOs water have higher *R_a_*^2^ values, but the ones for the stream and lake waters showed a relatively lower relationship for the measured TN and TP concentrations, probably due to their variability in properties of dissolved or particulate fraction. On the other hand, WWTP effluent and CSOs have relatively stable water quality compared with natural water; hence, regression models with higher *R_a_*^2^ values could be obtained. Figure 5 shows the variations of PO_4_–P and TP concentrations for each water type. While WWTP effluent and CSOs show relatively stable ratios between PO_4_–P and TP, the ratios of PO_4_–P to TP concentrations vary to some extent in stream and lake waters. This might be due to the possibility that particulate phosphorus was introduced from external sources into the stream and the lake.

**Figure 6:**
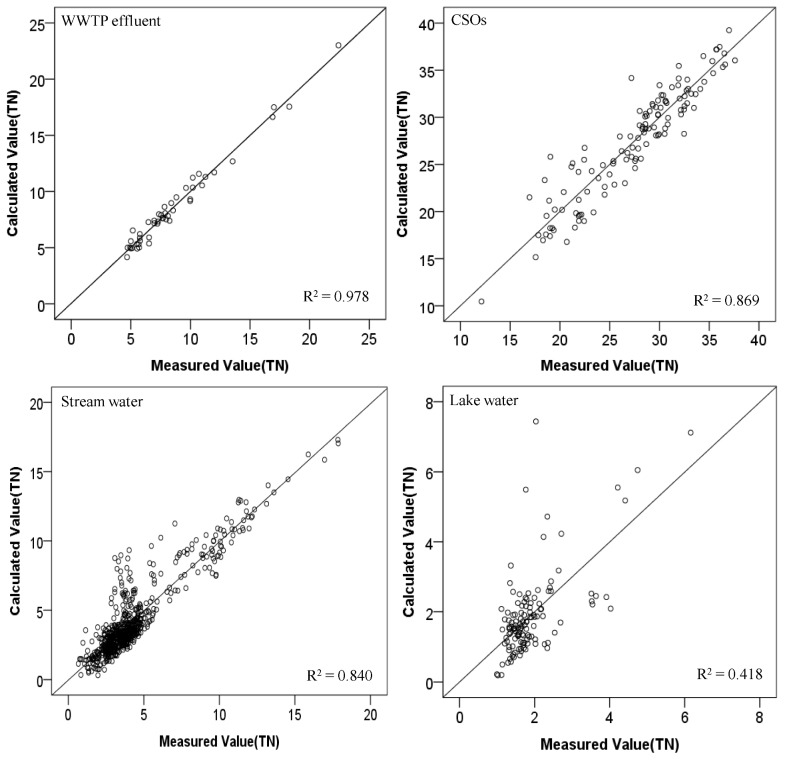
Comparison of measured and estimated TN concentrations for each water type.

**Figure 7:**
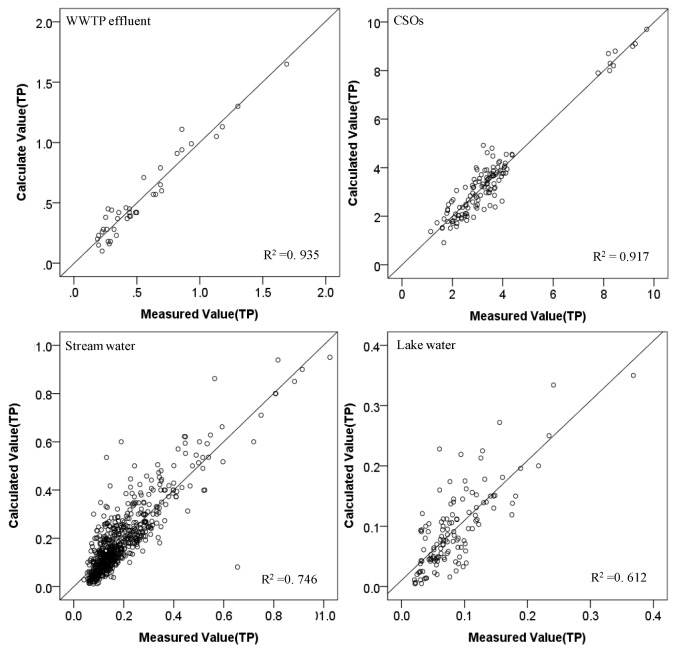
Comparison of measured and estimated TP concentrations for each water type.

Comparisons between measured TN or TP concentrations and those predicted by the software sensors for each water type were made in Figure 6 and Figure 7 for TN and TP, respectively. 

**Figure 8:**
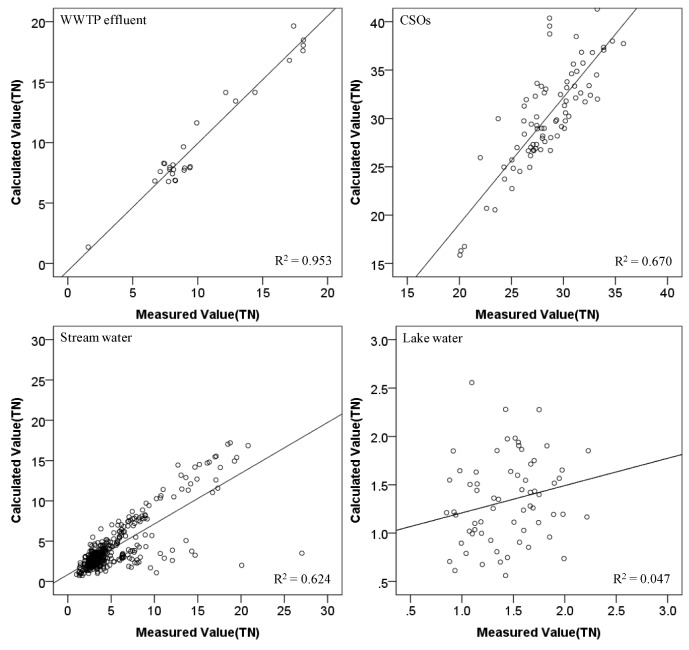
Validation of TN models for each water type.

**Figure 9:**
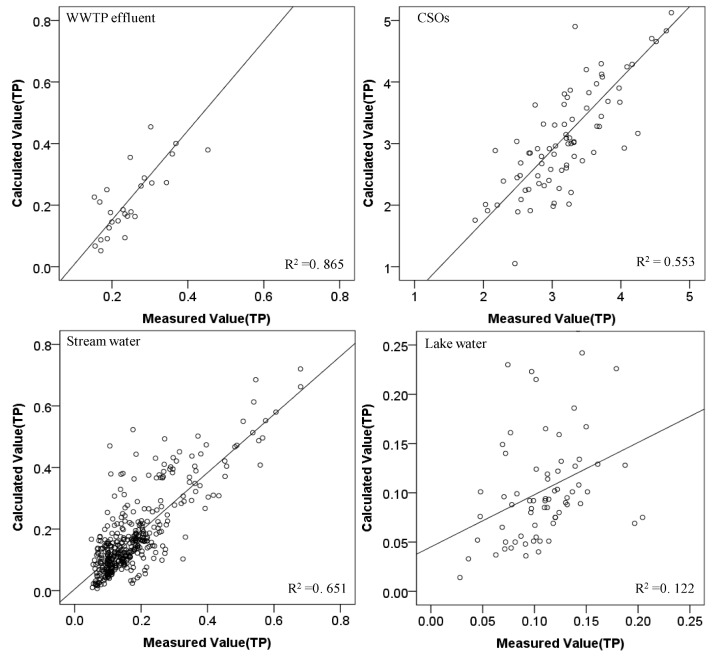
Validation of TP models for each water type.

For the validation of the developed models, the regression models were applied to another set of measured water quality data for each water type collected from September 2011 to June 2012. As shown in Figure 8 and Figure 9, the regression models developed in this study showed relatively good estimation for the WWTP effluent and CSOs. However, the ones for the stream and lake waters did relatively lower predictability. For streams and lakes, we would have obtained better results if we had calibrated the model for each season. In fact, we did not have enough data to do the seasonal analysis for the stream and the lake. In addition, most sampling stations for the stream and the lake had been frozen often during the winter season. If the ionic N and P species could be *in situ* monitored along with other physical parameters for river and lake waters in this study, and enough data could be obtained to utilize for model calibration within short period of time, we believe better predictions of TN and TP could be possible. 

**Figure 10:**
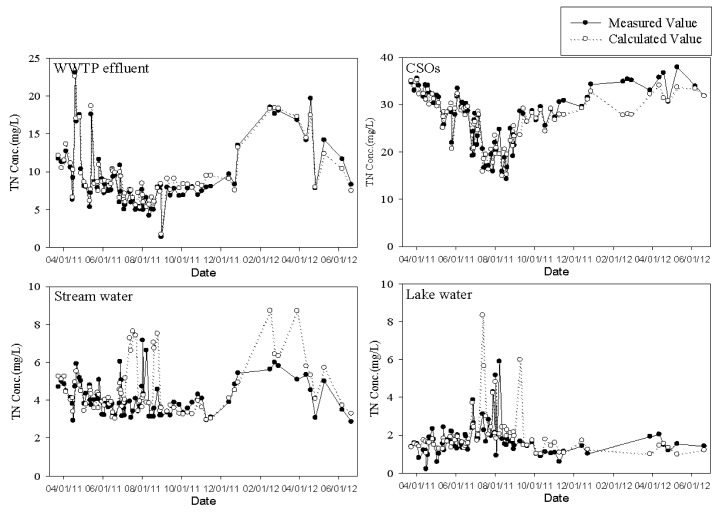
Time series of TN concentration predicted by software sensor.

**Figure 11:**
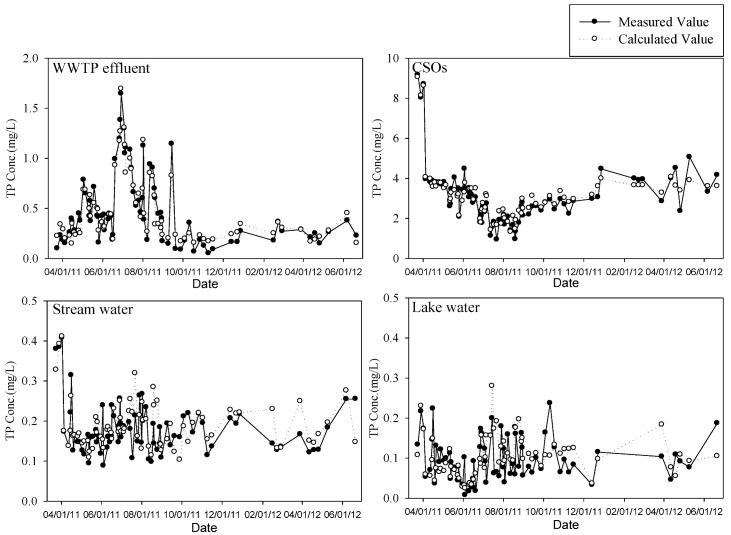
Time series of TP concentration predicted by software sensor.

Figure 10 and Figure 11 represent the time series of TN and TP concentrations estimated using the software sensors (or regression models) derived in this study along with measured data. As discussed above, the models follow the measured data well in the case of the WWTP effluent and the CSOs. In fact, the models for TN and TP of the stream and lake waters also reasonably follow the measured data except several points. If the time interval for data collection can be shortened, these intermittently occurring discrepancies between measured and predicted TN or TP values might be eliminated.

## 4. Conclusions

In this study, software sensors (or linear regression models) based on the MLR analysis algorithms were developed; they utilized other water quality parameters for predicting TN and TP concentrations of WWTP effluent, CSOs, stream water, and lake water. Initially, a few independent variables such as pH, DO, EC, Turb, NO_2_–N, NO_3_–N, NH_4_–N, and PO_4_–P concentrations were evaluated for their individual correlation with TN or TP; the variables with higher correlation with TN and TP were incorporated in the software sensors (or regression models) as an independent variables. 

In fact, the developed software sensors predicted the TN and TP concentrations for the WWTP effluent and CSOs waters reasonably well. In the case of the stream and lake waters, the predictability of the software sensors was relatively low, probably due to the low concentration ranges for the nutrients (especially for the TP) and variability of the ratios of PO_4_–P to TP concentrations due to the external influence to the water bodies, such as nonpoint source pollution or weather changes. 

From the result, nonetheless, it is expected that the proposed strategy (*i.e.*, application of a software sensor to monitor TN or TP) will allow the water researchers to monitor TN and TP in various water bodies more easily; especially for WWTP discharges and CSOs. If all the water quality parameters used as dependent variables for the regression models are analyzed *in situ* (as the case in the National Automated Water Quality Monitoring Program in Korea [20]), the software sensors for TN and TP can be easily realized and the two water quality parameters which are difficult to measure can be estimated continuously. 
